# Mood Changes by Self-Administered Acupressure in Japanese College Students: A Randomized Controlled Trial

**DOI:** 10.5539/gjhs.v7n4p40

**Published:** 2014-12-16

**Authors:** Satoshi Horiuchi, Akira Tsuda, Yasuhiro Honda, Hisanori Kobayashi, Mayu Naruse, Aki Tsuchiyagaito

**Affiliations:** 1 Iwate Prefectural University, Takizawa, Japan; 2 Kurume University, Kurume, Japan; 3 Fukuoka Tenjin Medical Rehabilitation Academy, Fukuoka, Japan; 4 University of Rhode Island, Kingston, United States; 5 Health Sciences University of Hokkaido, Sapporo, Japan

**Keywords:** college students, contemporary and alternative medicine, mood, self-administered acupressure

## Abstract

The aim of this 2-week study was to examine the effects of self-administered acupressure intervention onlevels of mood of 54 students (34 males and 20 females) majoring in acupuncture and moxibustion medicineat a college located in Fukuoka, Japan. Eligibility criteria were the ability to complete the intervention accurately and no history of psychiatric diseases. The students were randomly assigned to one of the two groups: an intervention group (IG, n = 28) and a control group (CG, n = 26). The IG participants completed fiveacupressure sessions three times a day (morning, noon, and night), involving the application of pressure to six acupuncture points (GB12, SI17, and LI18 according to 2008 World Health OrganizationRegional Office in the Western Pacific standard), three on the left and three on the right side of the neck for 5 s each. The CG participants were requested to spend their time as usual. Self-reported levels of tension-anxiety, depression-dejection, anger-hostility, vigor, fatigue, and confusion over the past week were measured before and after the study as the main outcomes. Side effects were not predicted and not assessed. The retention rate of this trial was 100%. Improvements in mood, defined as a change from baseline to 2 weeks later, were significantly greater in IG. Our results showed that self-administered intervention had the ability to alter mood levels in college students.

## 1. Introduction

In Japan, the management of mood levels is considered an important issue to maintain the mental health of students (Michimuko, Kinoshita, & Nishizawa, 1997). Katsuura et al. (2011) reported that 26.3% and 8.1% of 209 Japanese college students exhibited moderate to severe levels, respectively, of anxiety and depression. Therefore, from a mental health perspective, it is important to help Japanese college students to manage negative moods.

Acupressure plays an important role in both contemporary and alternative medicine (McFadden et al., 2012). According to the meridian theory of Chinese medicine, energy (Qi) flows through meridians, which are invisible circuitries or energy channels in the body. This theory assumes that mental or physical health is disturbed if the flow of Qi is too fast, too slow, turbulent, or static. Acupressure corrects the flow of Qi by applying pressure with the fingers to given acupuncture points. Importantly, acupressure can be self-administered to promote physical and mental health (Honda, Tsuda, & Horiuchi, 2012a).

Self-administered acupressure may be a useful self-care tool for college students to manage mood levels. Honda, Tsuda, and Horiuchi (2012b) reported that self-administered acupressure intervention, developed by Honda et al. (2012a), decreased the degree of depression–dejection, which was measured with the Japanese version of the *Profile of Mood States—Brief Form* (POMS-J; Yokoyama, 2005). The participants were instructed to complete five acupressure sessions three times a day (morning, noon, and night). Each session involved pressing six acupuncture points, three on the left and three on the right side of the neck for 5 s each. The three acupuncture points are known as GB12, SI17, and LI18, according to the World Health Organization Regional Office in the Western Pacific (2008). However, it remains unclear whether this self-administered intervention can reduce other negative moods, such as anxiety and anger, or improve positive moods in college students.

The aim of this study was to examine the effects of self-administered acupressure intervention (Honda et al., 2012a) of Japanese college students to provide new information on the ability of self-administered acupressure to alter mood levels. Specifically, the results of this study will help practitioners to use acupressure for the intervention of mood levels of college students.

## 2. Methods

### 2.1 Participants

A total of 54 college students (34 males and 20 females; mean age, 29.96 ± 9.10 years) majoring in acupuncture and moxibustion medicine at a college located in Fukuoka, Japan, were intentionally recruited for participation in this study for two primary reasons. First, these participants were trained to accurately locate the six acupuncture points used in the aforementioned study by Honda et al. (2012a). Secondly, the participants were a sample of convenience. One of the authors (Y. H.) was the instructor of several classes completed by the participants. The exclusion criterion was only a history of current or previous psychiatric illnesses; however, no participant fulfilled this criterion. The authors did not conduct a priori power analysis to determine an adequate sample size, as there has been no previous randomized controlled trial to examine the effect of self-administered acupressure on a variety of mood levels.

### 2.2 Outcome Measure

Mood levels were measured using POMS-J, which comprisessix mood scales: tension–anxiety, depression–dejection, anger–hostility, vigor, fatigue, and confusion (POMS-J; Yokoyama, 2005). Each subscale comprises five items. Participants were asked to report their mood levels over the past week on a 5-point Likert scaleranging from 0 (*not at all*) to 4 (*extremely*). POMS-J is a standardized tool of moods and its scores can be converted to T score [mean = 50, standard deviation (SD) = 10], using its manual (Yokoyama, 2005). This conversion is based on a large dataset, not on the study sample only. Thus, the mean and standard deviation of the participants were not necessarily 50 and 10, respectively. Higher T scores indicate higher levels of moods.

### 2.3 Procedure

The study protocol was approved by the Institutional Review Board of Kurume University. This study was conducted at a college located in Fukuoka prefecture, Japan, over a 2-week period in July, 2012. The participants gave an oral informed consent to participate in this study after receiving a thorough explanation of the study aims and procedure.A written consent was not obtained from each participant. One of the authors was a teacher to each participant, and some participants might hesitate to declare through written consent form that they would refuse to participate in this study. Effort was made to protect the right of each participant to refuse to participate in this study.

The participants were randomly assigned into one of the two groupsby one of the authors: an intervention group (IG) and a control group (CG). Finally, 28 students were assigned to IG and 26 to CG. The allocation was disclosed to the participants and the authors.

After completion of the classes, baseline mood levels were collected using POMS-J. The participants in IG were instructed to complete the self-administered acupressure intervention, as reported by Honda et al. (2012a), and perform five acupressure sessions thrice a day (on waking, after lunch, and before going to bed). For each session, the participants were instructed to apply pressure with the thumbs to the six acupuncture points (i.e., GB12, SI17, and LI18) for 5 s each for a sensation of comfort. These acupuncture points are located in the neck [for detail, see Honda et al. (2012a)]. The intensity of pressure to the accupoints was determined by each participant. One of the authors provided encouraging messages once a week to each participant in IG to effectively perform acupressure, while those in CG received no specific intervention. The participants in IG were not required to report whether they performed the acupressure procedure or not. After 2 weeks, mood levels were measured. The participants were followedup by numbers (e.g., 912), words (e.g., orange juice), or nicknames (e.g., little may) that they determined at baseline.

### 2.4 Analyses

Statisticalanalyses were performed using SPSS statistical software (version 22; IBM SPSS, Inc., Chicago, IL, USA). A probability (*p*) value < 0.05 was considered statistically significant.

## 3. Results

### 3.1 Baseline Characteristics and Mood Levels

IG comprised 20 men and 8 women, while CG comprised 14 men and 12 women. The results of the chi-squared test indicated that there was no significant difference in the male/female ratio between groups [χ^2^(1) = 1.79, *p* = 0.18]. The average (± SD) participant age was 32.07 (8.02) and 27.69 (7.51) years in IG and CG, respectively. The results of the unpaired *t*-test indicated significant differences with regard to the average age of participants between groups [t(52) = 2.07, *p* = 0.04].

Table 1 summarizes the mean (± SD) scores of the six subscales at baseline and after 2 weeks. The baseline mood levels of both groups were below or around 50 points. These results indicated that the mood status of the two groups was considered average or better. Six unpaired *t*-tests showed that both groups were fairly well matched with regard to baseline mood levels [tension–anxiety, t(52) = 0.49, *p* = 0.63; depression–dejection, t(52) = 0.64, *p* = 0.52; anger–hostility, t(52) = 0.1.26, *p* = 0.21; vigor, t(52) = 0.92, *p* = 0.36; fatigue, t(52) = 0.30, *p* = 0.76; confusion, t(52) = 0.75, *p* = 0.46].

**Table 1:** Outcome measures for IG and CG at baseline and after 2 weeks

	Mean (SD)			

	Intervention (n = 28)	Control (n = 26)	*F*	*η*^p2^
Tension–anxiety				
Baseline	47.39 (9.65)	48.81 (11.63)		
After 2 weeks	43.39 (9.22)	50.38 (13.16)		
Improvement	−4.00 (4.52)	−1.58 (9.03)	7.83**	.14
Depression–dejection				
Baseline	47.79 (10.07)	49.65 (11.18)		
After 2 weeks	45.21 (7.78)	51.69 (11.97)		
Improvement	2.57 (4.00)	−2.04 (4.72)	16.81**	.25
Anger–hostility				
Baseline	45.71 (8.67)	42.96 (7.25)		
After 2 weeks	42.61 (6.71)	45.38 (9.65)		
Improvement	3.11 (5.31)	−2.42 (8.08)	10.60**	.18
Vigor				
Baseline	44.29 (13.68)	47.54 (12.33)		
After 2 weeks	47.50 (13.28)	43.65 (13.22)		
Improvement	3.21 (7.99)	−3.88 (9.19)	7.88**	.14
Fatigue				
Baseline	51.86 (12.10)	50.88 (11.53)		
After 2 weeks	47.46 (9.42)	52.54 (11.50)		
Improvement	4.39 (6.76)	−1.64 (6.75)	9.52**	.16
Confusion				
Baseline	48.71 (9.60)	51.08 (13.33)		
After 2 weeks	45.68 (8.48)	51.73 (13.49)		
Improvement	3.03 (4.55)	−0.64 (5.61)	7.97**	.14

** *p* < 0.01; SD, standard deviation.

*Note*. F values for age and the baseline mood scores were not showed.

### 3.2 Retention Rates

All of the participants completed the study, thus the participation rate was 100%. This very high participation rate may be because the participants’ majors were close to the study topic. No one dropped out from the trial, thus the retention rate was 100%. This very high retention rate may be because the participants could complete the questionnaires while attending classes.

### 3.3 Intervention Efficacy

For each negative mood, score improvement was calculated for each participant by subtracting the score 2 weeks later from the baseline score. For vigor, score improvement was calculated for each participant by subtracting the baseline score from the score at 2 weeks. A greater value in score improvement indicates greater improvement in mood level. Table 1 also indicates improvement scores for the six subscales. For each mood, analysis of covariance (ANCOVA) was conducted for each group (intervention vs. control) as the independent variable, the baseline score and age as covariates, and each mood score as the dependent variable.

ANCOVA detected significant effects for tension–anxiety (F(1, 50) = 7.83, *p*< 0.01), depression–dejection [F(1, 50) = 16.81, *p*< 0.01], anger–hostility [F(1, 50) = 10.60, *p*< 0.01], vigor [F(1, 50) = 7.88, *p*< 0.01], fatigue [F(1, 50) = 9.52, *p*< 0.01], and confusion [F(1, 50) = 7.97, *p*< 0.01]. These results indicated that participants in IG achieved significantly greater improvements than those in CG. To interpret the magnitudes of these effects, values of *η*^p2^ were presented in Table 1 for the six subscale scores. Partial *η*^p2^ ranged from .14 to .25. These effects were within a large range according to the Cohen (1988) guidelines for interpreting effect size. The score improvements were negative in CG, showing that CG participants, on average, showed increased levels of negative moods and decreased levels of positive mood (i.e., vigor). Such mood disturbances in CG may partially contribute to the large effects.

## 4. Discussion

This study is one of the first to demonstrate that self-administered intervention can decrease levels of tension–anxiety, depression–dejection, anger–hostility, fatigue, and confusion, and increase vigor. These results were consistent with those of Honda et al. (2012b), who reported that self-administered acupressure intervention significantly reduced the severity of depression. By showing that this intervention also altered other types of negative moods (i.e., tension-anxiety, anger-hostility, fatigue, and confusion) and positive mood (i.e., vigor), the results of this study extended existing knowledge.

Self-administered acupressure, as described by Honda et al. (2012a), was shown to effectively improve a variety of moods of college students. This new evidence is important in Japan where managing mood levels is an important health issue among students. The acupressure intervention described here can be self-administered once students learn where and how to apply pressure to the six acupuncture points. Practitioners in the field of school healthcare can select from a variety of methods to assist college students with mood regulation. For example, the effectiveness of several relaxation techniques, such as autogenic training, progressive relaxation, and biofeedback, has already been demonstrated. However, no technique has been reported to be effective in all individuals. Thus, additional choices of methods to manage mood will aid in the design of interventions according to an individual’s needs (Honda et al., 2012a).

Although this study provided new evidence supportive of the ability of self-administered acupressure to reduce mood levels, the following two limitations should be addressed. It is unclear whether these findings can be generalized to other student populations. First, this study focused on college students who are majoring in acupuncture and moxibustion medicine. These students seem to be more likely to be interested in acupressure, and this may contribute to very high participation and retention rates. Second, this study lacked a placebo-control group. Based on this study, it is required to conduct another randomized controlled trial wherein such a control group is established. Therefore, it is necessary to examine the generalizability of the findings.
